# Computational fluid dynamic models as tools to predict aerosol distribution in tracheobronchial airways

**DOI:** 10.1038/s41598-020-80241-0

**Published:** 2021-01-13

**Authors:** Claudia Atzeni, Gianluca Lesma, Gabriele Dubini, Maurizio Masi, Filippo Rossi, Elena Bianchi

**Affiliations:** 1 Laboratory of Biological Structures Mechanics (LaBS), Department of Chemistry, Materials and Chemical Engineering “Giulio Natta”, Politecnico Di Milano, Piazza Leonardo da Vinci 32, 20133 Milan, Italy; 2 Department of Chemistry, Materials and Chemical Engineering “Giulio Natta”, Politecnico Di Milano, Piazza Leonardo da Vinci 32, 20133 Milan, Italy

**Keywords:** Anatomy, Health care, Medical research

## Abstract

Aerosol and pollutants, in form of particulates 5–8 μm in main size face every day our respiratory system as natural suspension in air or forced to be inhaled as a coadjutant in a medical therapy for respiratory diseases. This inhalation happens in children to elderly, women and men, healthy or sick and disable people. In this paper we analyzed the inhalation of aerosol in conditions assimilable to the thermal therapy. We use a computational fluid dynamic 3D model to compute and visualize the trajectories of aerosol (3–7–10–25 µm) down to the sixth generation of bronchi, in a steady and dynamic condition (7 µm) set as breath cycle at rest. Results, compared to a set of milestone experimental studies published in literature, allow the comprehension of particles behavior during the inhalation from mouth to bronchi sixth generation, the visualization of jet at larynx constriction and vortices, in an averaged characteristic rigorous geometrical model including tracheal rings. Results on trajectories and deposition show the importance of the including transient physiological breath cycle on aerosol deposition analyses. Numerical and graphical results, may enable the design of medical devices and protocols to make the inhalations more effective in all the users’ population.

## Introduction

Understanding particle deposition is important in risk assessment for toxic air pollutants as well as in evaluation of the efficacy of therapeutic aerosols. The complex lung morphometry, the cyclic unsteady nature of the respiratory flow, air and particle properties have major effects on trajectories and deposition of inhaled particles^1^. The use of computational fluid dynamic (CFD) tools allows the robust description and prediction of transport and deposition of particles in a defined 3D structure, based on mathematical models. The analyses and the visualization of complex phenomena in a 3D accessible format is a determining tool for clinical decisions, designing biomedical devices and clinical protocol, and in explaining phenomena for educational purposes. First step of the computational approach is the definition of a geometrical model. Many models have been created to investigate airflow patterns and particle transport in the respiratory airways through experimental and computational approaches^2^. According to Walenga et al.^3^, such models can be categorized as simplified, patient-specific and characteristic models. Simplified models are geometrically similar to the respiratory airways, but miss features that could affect quantitative outputs. Patient-specific models are obtained as reconstructions from in-vivo images and they are able to correctly reproduce all the phenomena occurring in an individual subject. A characteristic model, as the one described in this paper, reproduces all geometrical features of an average subject while neglecting unnecessary patient-specific details that could decrease the predictive efficiency of the model. Considering the domain extension, these models can be further categorized as single^4^, double^5^, or multiple^6,7^ bifurcation models, complete tracheobronchial (TB) tree models^8–10^, upper airways models^11–13^, and complete airways models^3,14–20^.

The most used simplified model was created by Weibel et al. and consists of 23 generations of symmetric bifurcating airways^21^, defined by their lengths, diameters and branching angles between parent and daughter branches. Based on Weibel data, Balàshàzy et al. proposed a model of realistic asymmetric bifurcation where the parent and daughter tubes do not lie on the same plane and are delimited by a transition zone, which contains the two curved portions of daughter tubes^4,22^. Similarly, Heistracher et al.^23^, defined a mathematical method to generate a 3D asymmetric bifurcation model with a smooth surface through the definition of the radius of curvature and the carinal ridge. In order to achieve a precise model of the human TB tree, in-vitro preparation of lung casts from cadavers can be combined with high resolution computed tomography imaging (CT) technique. The most widely used model is the digital reference model proposed by Schmidt et al.^8^, which contains 1453 bronchi extending down to the 17th generation. With regard to the *upper airways*, a number of studies tried to assess the role of upper morphometry on particle deposition. Using measurements from CT scans, magnetic resonance imaging and direct observation of patients, they describe idealised models of the extra thoracic airways with simple geometric features, reproducing mouth, pharynx, larynx and trachea. Cheng et al.^11^ investigated characteristic shapes, perimeters and cross sectional areas (CSA) of human upper airways extending from the oral cavity to the upper trachea. CSA varies along the model, with a minimum of 0.64 ${\mathrm{cm}}^{2}$ in proximity of the glottis. Similarly, Stapleton et al.^12^, created an average model of *extra-thoracic airways where both pharynx and glottis* are simplified as elliptical tubes with CSA equal to 2.98 ${\mathrm{cm}}^{2}$ and 0.95 ${\mathrm{cm}}^{2}$, respectively. Starting from Cheng model^11^, Xi et al.^13^, introduced realistic features such as the physiological airway curvature, a half-mouth opening, a triangular-shaped glottis and the 17° dorsal angle. A model involving all pathway from the first bifurcation to the sub-acinus unit, by means decomposition and reduction of complexities of the TB, is the one presented by Koullapis^24,25^. Recently, complete models of human airways comprising both upper airways and TB tree down to the 6th–7th^3,14,15,18,19^, or to the 17th^26^, generations have been realised.

On these reconstructed 3D airways models it was possible to applied numerical methods and perform CFD simulations of airflows in flowing and depositing particles. The 3D model of interest has to be discretized by the definition of a grid, whose quality and topology influence the quality and the robustness of the resulting data. Flow is computed by means of numerical models of the Navier–Stokes equation. The effects of air fluid dynamics and particle properties can usually be assessed considering various combinations of Reynolds and Stokes numbers^3,15,20,27–31^. Reynolds is a dimensionless number, ratio between inertial and viscous forces, used to define the laminar/turbulent regime of flow. Stokes is another dimensionless number defined as the ratio between the characteristic response time of the fluid and the characteristic response time of the particle, used to characterize the ability of particles to follow the fluid streamlines^32^. The transport and deposition of particles, intended to represent pollution or aerosol particles, are investigated by means of dedicated numerical models, interacting or consequent to the airflow models. Particle deposition is commonly evaluated in terms of particle deposition efficiency (DE), defined as the dimensionless ratio of the number of particles deposited in a given region to the total number entering the region^5,7,12,13,17,28,29^.

Accurate steady state simulations, defined as the representative condition in airways leading to particle deposition, were described in the several works of the scientific literature. Comer et al.^5^, numerically simulated deposition of inhaled 3–7 µm particles in a 3D double bifurcation model, assuming a steady, laminar and constant-property airflow. They observed a variation in deposition patterns with the bifurcation level and orientation. The distribution was symmetric near the carina of the 1st bifurcation, while it appears very asymmetric at the 2nd generation walls. Similarly, Zhang et al.^33^ extended this analysis down to the 3rd bifurcation, simulating spherical non-interacting particles. Gemci et al.^9^ performed simulations by CFD to investigate airflow pattern in Schmidt^8^ 17-generations model. A constant airflow rate of 28.3 L/min was imposed to simulate the turbulent regime using a large eddy simulation (LES) turbulence algorithm. With an airflow rate ranging from sedentary to normal breathing conditions, they observed the development of secondary patterns in the 1st generation, well as the increase of the viscous pressure drop from 6 Pa at 6 L/min to 54 Pa at 30 L/min^9^. Xi et al.^13^ imposed a constant airflow rate of 30 L/min to the realistic featured model, to simulate a turbulent airflow using the low Reynolds number (LRN) k–ε model. A laryngeal jet appeared upstream the glottis region due to the reduced CSA and produced recirculation zones near the trachea. It was observed that the laryngeal jet combined with a physiological trachea dorsal angle enhanced particle deposition around the glottis and the upper trachea.

Lambert et al.^15^ investigated regional deposition of 2.5–30 µm particles in a CT-based human airway model down to the 7th generation. The turbulent flow in the upper respiratory tract was simulated with LES imposing a steady-state parabolic airflow of 20 L/min at the inlet. Particle deposition increased with increasing particle size and in proximity of bifurcations, due to the inertial effects. The filtering effect of the oral cavity was very pronounced for 30 µm particles, while DE of 10 µm particles was more uniform in spite of high concentration in larynx. They also highlighted that the left lung received a greater portion of the particles with respect to the right lung. Similarly, Rahimi-Gorji et al.^19^ analysed airflow and particle deposition under various breathing conditions in a realistic airway model down to the 6th bronchus generation, obtained as a reconstruction from CT scans. Steady-state CFD simulation were performed to solve the flow field while spherical/inert particle deposition was simulated using the discrete phase model (DPM). At higher flow rates (> 30 L/min) the region between pharynx and larynx showed maximum velocity near the outer wall due to centrifugal forces, thus trapping large diameter particles. On the other side, in a laminar regime, the lower inertial forces directed more particles deeper up to the 4th–5th generations. In Kim et al.^7^ the results of simulation on a new realistic parametrically controlled airway model, down to the 11th generation, found a good agreement to Weibel model.

More recent advancements in the simulation tools and the increasing of the IT calculation capacity have led to the design of more ambitious models, involving a deeper featuring of the anatomical and respiratory details. The lung model of Elcner et al.^14^ contains realistic geometries for the throat, the trachea and the TB tree up to the 4th generation. Data of upper airways were acquired from CT scans of living subjects, while the reconstruction of TB tree was based on Schmidt data. Experiments and transient CFD simulations were carried out to reproduce a complete sinusoidal breathing cycle under both sedentary (7.5 L/min) and deep breath (15 L/min) regime. A laryngeal jet clearly formed and evolved during the breathing cycle and its effect propagated downstream the trachea with the formation of separation zones in both main bronchi. Calmet et al.^18^ performed large-scale CFD simulation on a highly-detailed geometry of lung down to the 4th bifurcation in order to explore the airflow dynamics during a rapid inhalation cycle. A dominant turbulent flow was observed along the descending airways due to the strong laryngeal jet, which quickly dissipated with the breakup of vortex structures. Walenga et al.^3^ compared data from simplified and realistic models of TB tree down to the 3rd generation with the empirical data of Zhou et al.^28^ to quantify the influence of geometrical features (i.e. tracheal curvature, main bronchi curvature and irregular cross sections) on pharmaceutical aerosol deposition. They found that accurate tracheal features enhanced regional deposition and shifted particle patterns towards left walls. Realistic CSAs and bifurcation curvature increased DE in the TB tree^3^. Moreover respiratory air flow characterized in the lung is nonsymmetrical while the pattern of flow field was similar.

Das et al.^20^ used an idealized, anatomically-faithful upper airway geometry, where simulations are function of age, from a 5 year old to an adult. The results of the comparison between a Dry Powder Inhalers (DPI) and nebulizer inhalation performance were collected in a dimensionless curve governing deposition in the airways via Stokes number. A patient-specific model was then simulated by Farghadan et al.^10^ within a breathing cycle, and the use of Finite-time Lyapunov exponent (FTLE) was involved to compute the Lagrangian topological maps that define the destination of particles at the trachea. Tian et al.^16^ compared steady state and transition simulations of particles deposition in a characteristic patient model, by means of a Stochastic Individual Path (SIP) model in branches deeper than TB4.

CFD results were compared for validation to experimental data on total and regional DE. Experimental data were obtained from both human volunteers and airway hollow casts^28–31,34–37^. For example Chan and Lippmann^31^ studied the regional DE in a hollow cast of the human larynx and TB tree extending down to the 6th generation, and in 26 human volunteers, finding a linear dependence of particle DE on Stokes number for aerosols with aerodynamic diameters greater than 2 µm.

Similarly, Cheng et al.^29,34^, investigated the effects of particle size and breathing conditions on DE in a human oral airway replica. They observed that DE increased with higher flow rates and particle diameters, suggesting that impaction is the dominant deposition mechanism. Furthermore, by comparing their results with in-vivo deposition data^31,37^, they defined a relationship between DE and the impaction parameter IP, defined as:

1 $$\mathrm{IP}={\uprho {d}_{p}}^{2}\mathrm{Q}$$

where ρ is the density of the particle, ${d}_{p}$ is the aerodynamic diameter and *Q* is the flow rate^29^. Regional DE in a TB tree replica was carefully studied by Zhou et al.^28^ through the injection of fluorescent particles at constant flow rates (15, 30, 60 L/min). They observed a relationship between DE and Stokes numbers, bifurcation angles and diameters and provided an empirical model to estimate DE in each generation.

Many partial/complete model of human airways were create to investigate airflow pattern and particle DE under various breathing conditions. Airflow pattern was widely analysed under both constant flow rates^5,9,13,15,26^, and transient breathing cycle^14,18,38^, while most of particles analyses found in the literature were performed under constant airflow conditions. Gurman et al.^36^ suggest that this approximation can represent large particle cases pretty well while it underestimates total DE of smaller particles.

Only a few recent works analysed the behaviour of nanometric and micrometric particles during a complete breath cycle^10,16,24,38^, under different breathing conditions (sedentary, light and heavy breathing).

The focus of the present research is to create a characteristic model of human airways, in order to investigate therapeutic aerosol DE, including the transient behaviour of a physiologically breathing cycle in sedentary conditions. A deep analysis of the anatomical features described in literature suggested the involvement of a special strategy to design the geometry of bronchi around bifurcations and the introduction of the cartilaginous rings in the reconstruction of the trachea. Presence of rings, usually considered as structural components, modifies the appearance of the tracheal wall as sub-millimetric features^16^ that may have a role in the formation of boundary layer of flow and in the deposition of particles, consistently with in-vitro observations of Russo et al.^39^. The model of human airways has been constructed to allow future scalability based on the age-specific anatomy.

## Results

Results of numerical simulations are tools to visualize and describe the behaviour of air and aerosol along a respiratory cycle.

### Airflow

**Figure 1:**
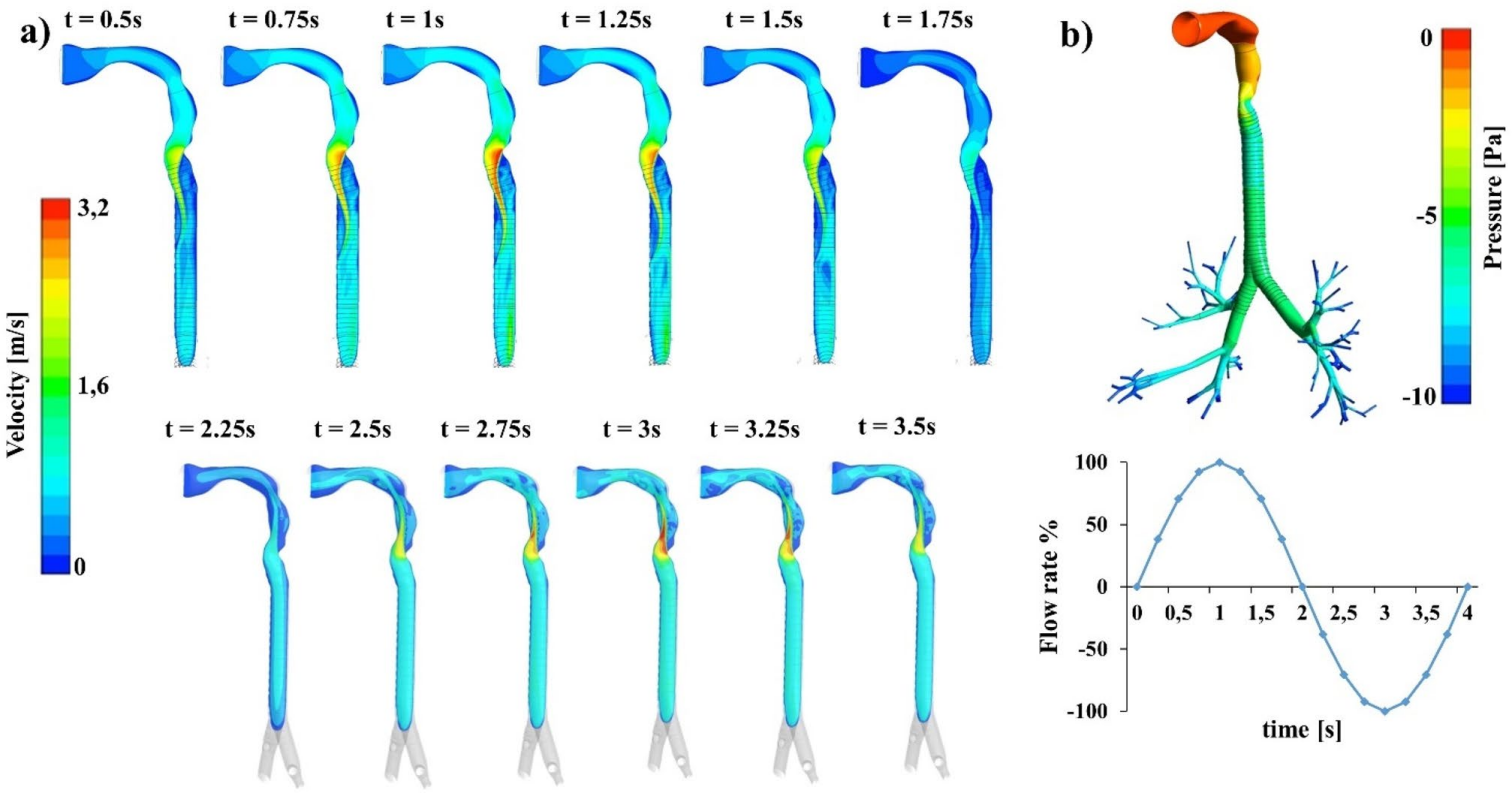
Airflow pattern and pressure trend during sedentary breathing cycle (**a**) Magnitude of velocity on the longitudinal plane. In evidence the acceleration across the larynx constriction. (**b**) Static pressure field along the respiratory system at the inspiration peak (t = 1 s). (Fluent v16, CFD-Post v16-Ansys, www.ansys.com).

Ventilation was quantified considering the airflow exiting the RUL (right-upper lobe), the RLL (right-lower lobe) and the RML (right-medium lobe), while left ventilation took into account the LUL (left-upper lobe) and the LLL (left-lower lobe) outflows. Reasonable consistency of the percentage ventilation with published data^40–42^, was found for each lung lobes: RUL 19%, RLL 29%, RML 9%, LUL 26% and LLL 17%. The total left and right total ventilation values were 43% and 57%, respectively. The resultant distribution is translated to the transient simulation, by means of dedicated script. The velocity profile at each cross section varies continuously during one complete cycle of respiration, depending on cross sectional areas, the geometrical features and the velocity profile at the inlet of the region (Fig. 1a)^1^. As expected, a laryngeal jet appears in the glottis region, where the noticeable constriction of the larynx forces the airflow to accelerate^16,20^. The strong centrifugal forces shift this high velocity region towards the posterior wall during the inhalation phase (0–2 s), while the peak is shifted towards the anterior walls during the exhalation phase (2–4 s). Nevertheless, airflow always exhibits the characteristics of laminar or quasi-transitional flow, reaching a maximum Reynolds number equal to $\mathrm{Re}=1848$ at $\mathrm{t}=1\mathrm{ s}$ in the glottis, which is the most critical section in terms of area and local velocity. The absence of a turbulent regime is reasonable considering the sedentary breathing condition: turbulent phenomena appear for airflow greater than 10–12 L/min^1,9,14,26^.

The static pressure distribution in Fig. 1b shows a pressure drop equal to 4.5 Pa between the tracheal inlet and the 6th branches at the inspiratory peak, which is close to measurement by Gemci et al.^9^.

**Figure 2:**
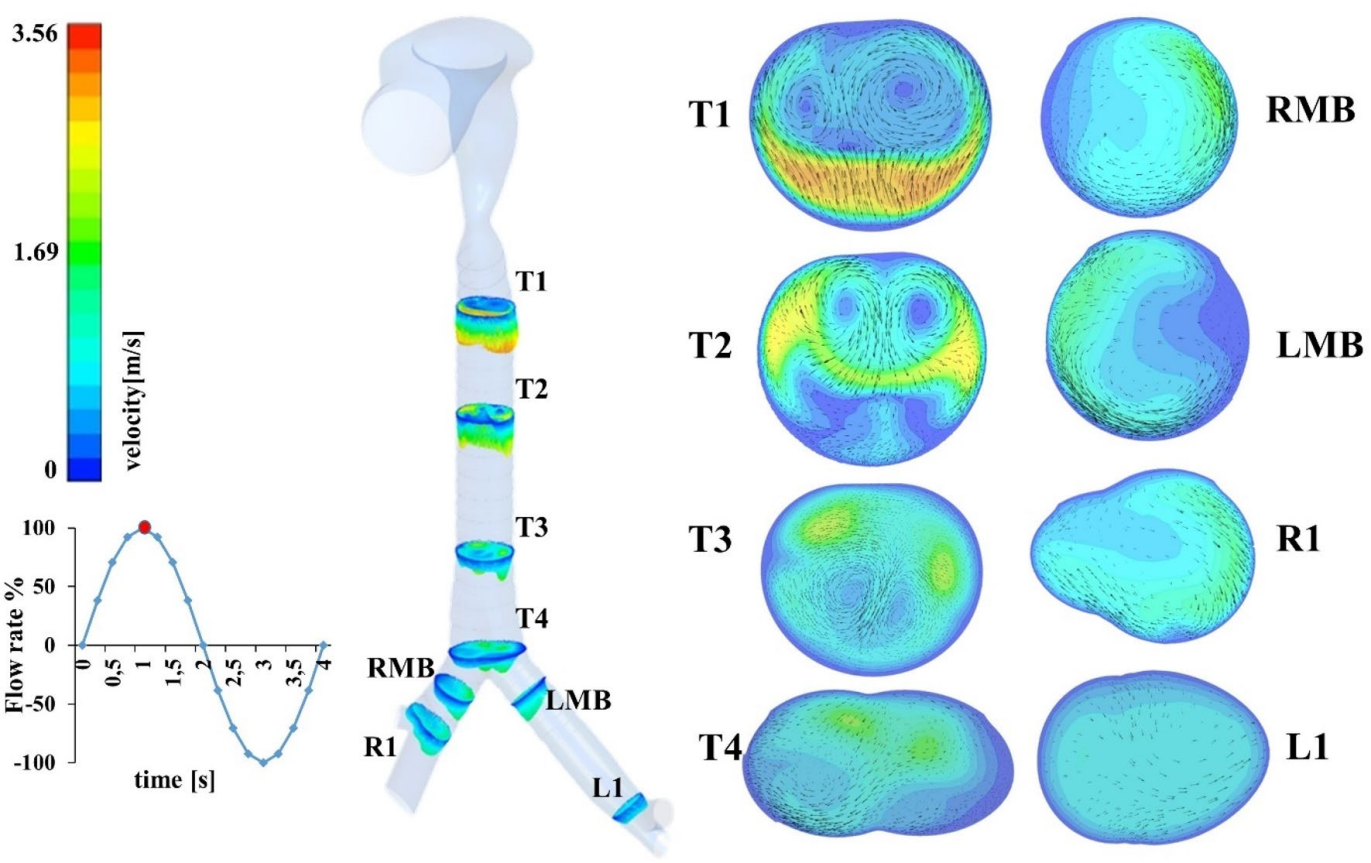
Development of the velocity field downstream the trachea at *t* = 1 s, in terms of velocity magnitude and tangential component of the velocity vectors. In evidence the rotational flow on the plane of proximal trachea, decreasing in the distal section down through the bifurcation. (Fluent v16, CFD-Post v16-Ansys, www.ansys.com).

The laryngeal jet effect propagates downstream the trachea with the development of secondary structures in the radial direction (Fig. 2).

**Figure 3:**
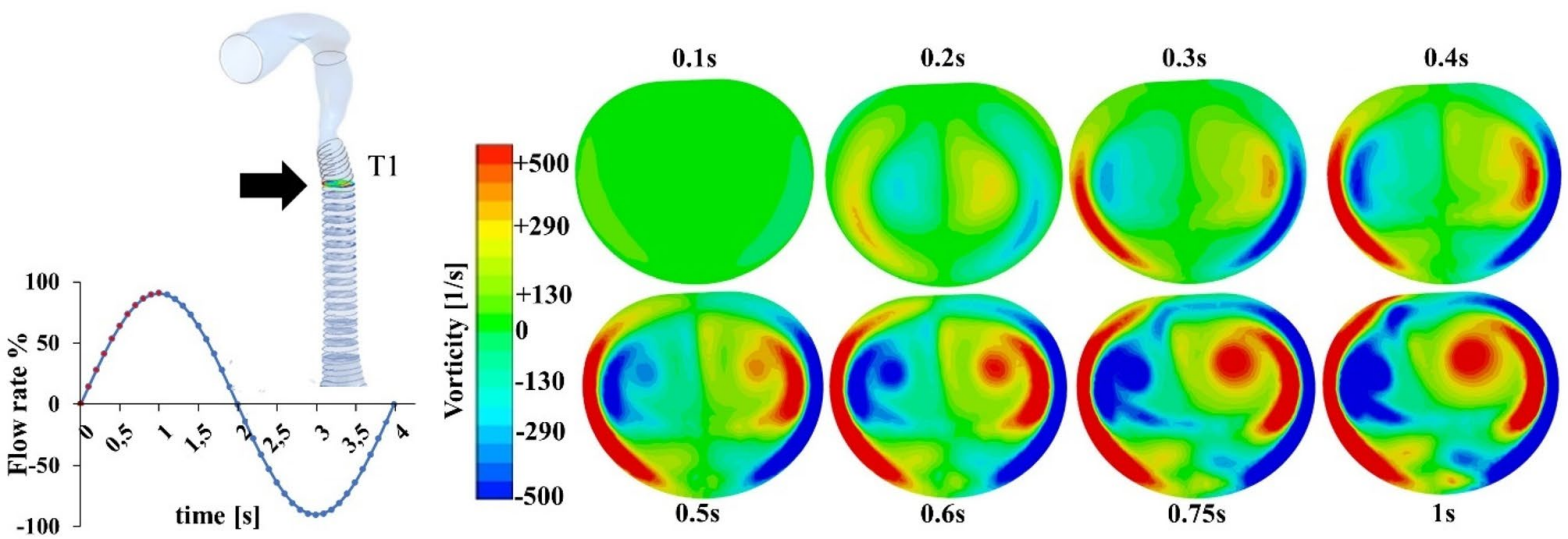
In evidence the asymmetrical development of the vorticity field at cross section T1, reaching the maximum at the end of the ascendant part of the inhalation cycle (0–1 s). (Fluent v16, CFD-Post v16-Ansys, www.ansys.com).

The unbalance between the anterior fast and directional glottides jet and the posterior still air gives rise to double and opposites eddies. These recirculation zones were further investigated in terms of vorticity. At *t* = 0.2 s two opposite vortices appear near the dorsal wall of section T1 (Fig. 3), then they progressively develop until they reach a maximum value of vorticity equal to 500/s at the inspiratory peak (*t* = 1 s). Each vortex core is progressively shifted towards the anterior wall (T3) downstream of the trachea, while the intensity decreases. In the proximity of the 1st bifurcation, the main bronchi velocity profile (LMB, RMB) shows the peak near the inner walls, which is confirmed by other works^1,9,14,43^. Airflow pattern in downstream branches is regular and symmetric, due to the highly-laminar regime of these regions. The presence of transversal fluid velocity affect the spatial distribution of particles towards trachea wall.

### Particulate phase

**Figure 4:**
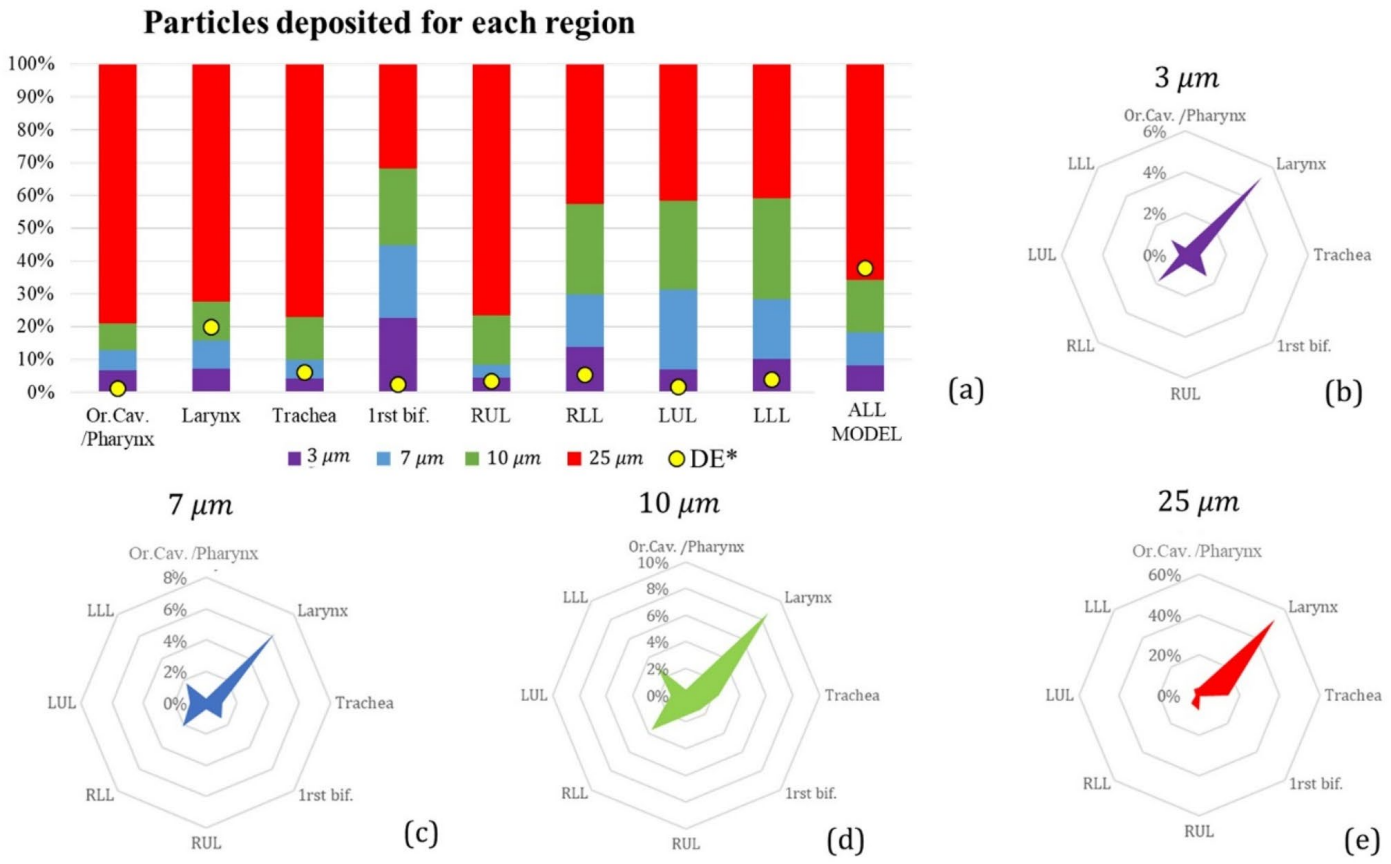
(**a**) Size distribution of particles (3–7–10–25 µm), as fraction of deposited particles in each region under steady conditions (Q = 6 L/min). DE* is total deposition efficiency for each region and for all models. RUL and RLL are the right upper and lower lobes, LUL and LLL are the left upper and lower ones. RML have been included in RLL. (**b**–**e**) DE made explicit for each simulated size in every region.

Steady DE (Eq. 8) of 3–25 µm particles in the oral cavity, larynx, trachea, 1st bifurcation and the four lobes are listed in Fig. 4. Increasing particle size, thus particle inertia, the filtering effect of larynx increases. DE of 7–10 µm particles is more uniform although a high concentration correctly persists in the larynx^15^ (see “Supplementary Materials”, Table S1). Generally, the right lung receives a greater portion of the particles, which is reasonable considering the greater flow ventilation in this region and the laminar regime simulated (< 10 L/min)^19^.

**Figure 5:**
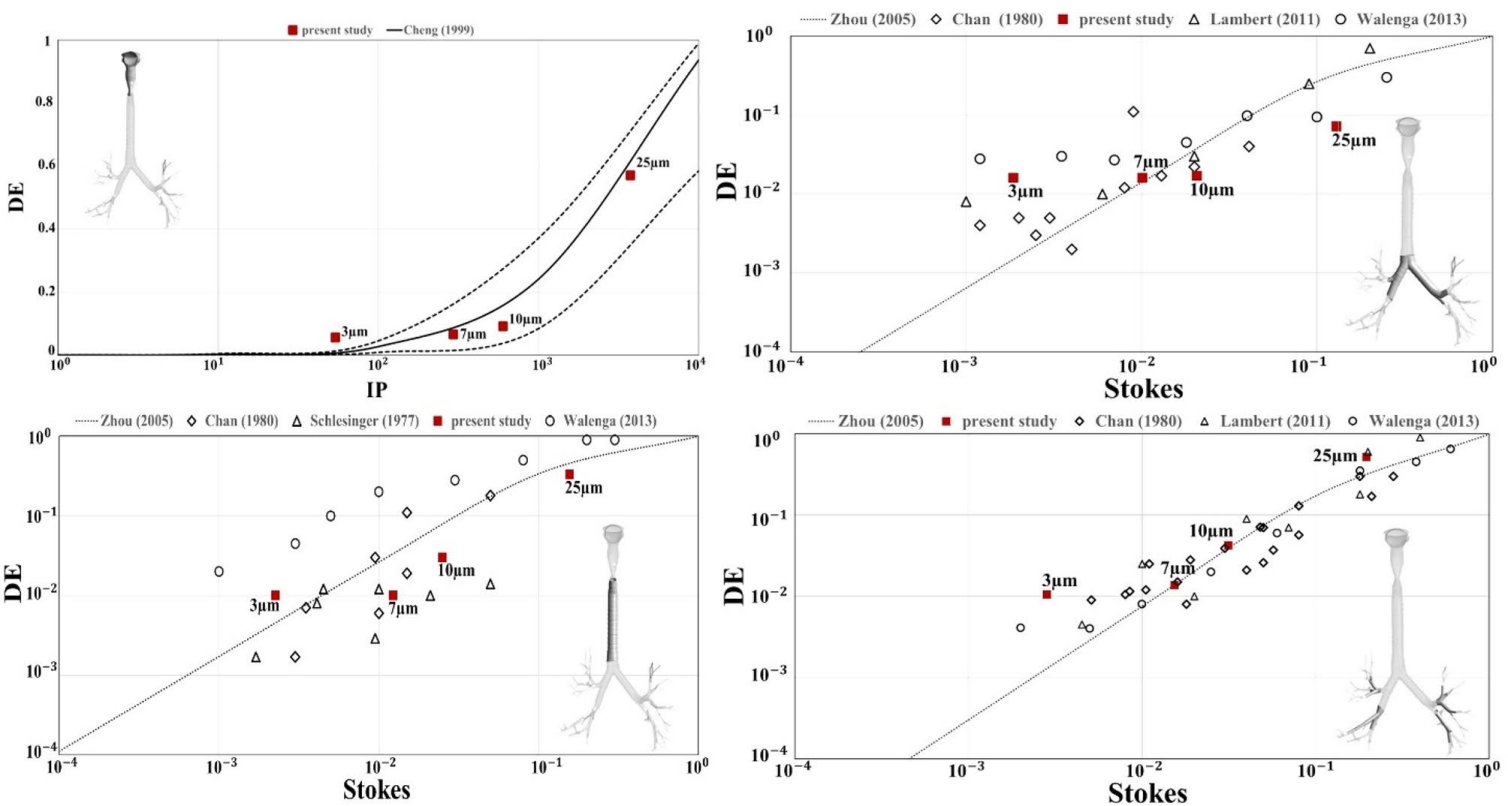
Deposition efficiency (DE) of 3–25 µm particles injected under steady conditions (Q = 6 L/min). RUL and RLL are the right upper and lower lobes, LUL and LLL are the left upper and lower ones. DE in the upper airways is presented as a function of the impaction parameter IP (Eq. 1), while the Stokes number (Eq. 7) better represents the DE behaviour in downstream regions.

Under steady-state conditions, regional DE of 3–25 µm particles is shown in Fig. 5, respectively, for validation purposes against data in the literature.

DE of our characteristic model showed a similar trend as the empirical model of Zhou et al.^28^ and other data found in the literature^3,15,29–31^. Good agreement between the empirical formulation and the characteristic model data was achieved with 7 µm particles, especially at the 1st and 2nd bifurcations.

**Figure 6:**
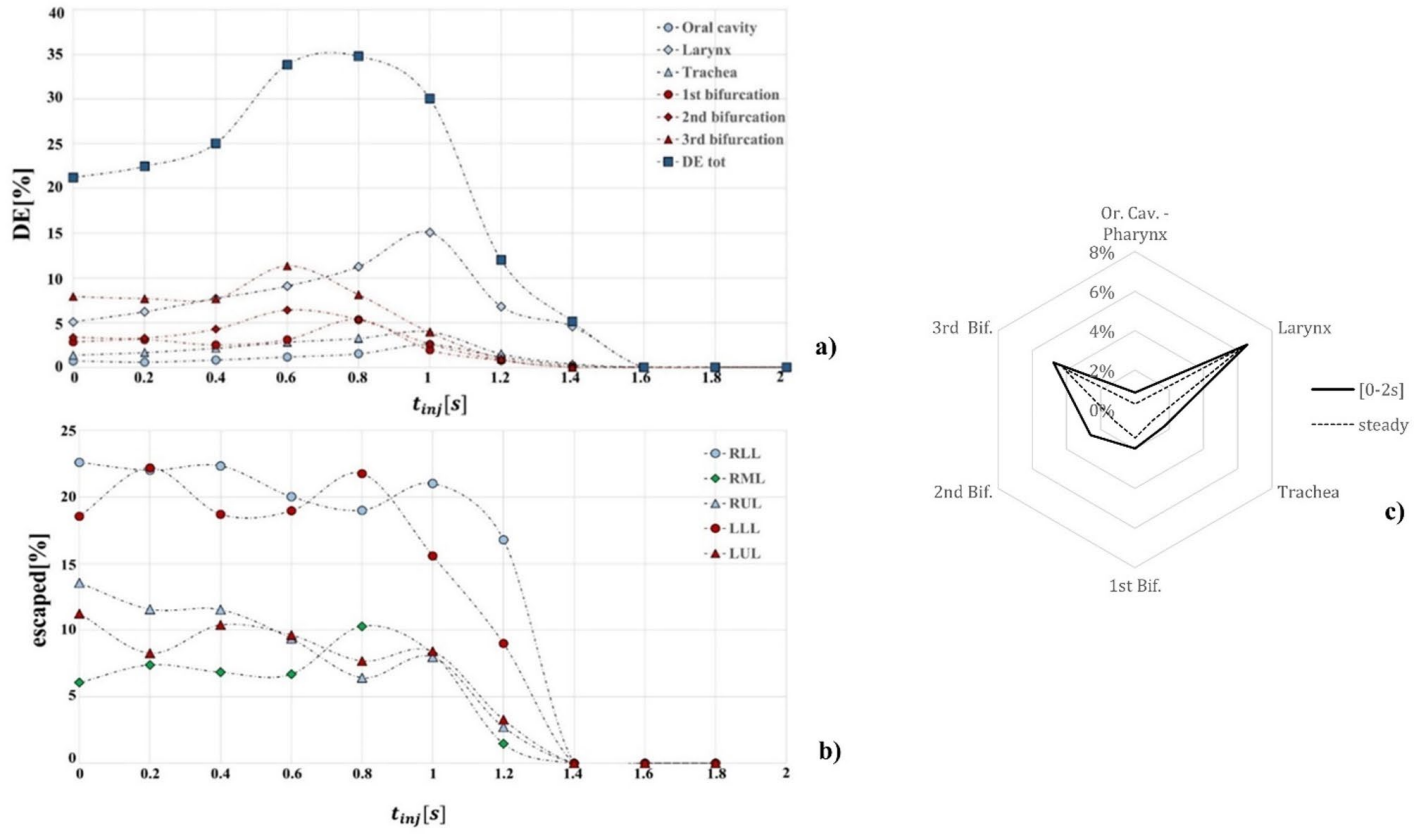
(**a**) Total deposition efficiency (DE) of 7 µm particles in different regions of the model for various injection times; (**b**) percentage of particles escaped from the different lobes of the lung model for various injection times, (**c**) total DE in steady and unsteady simulations, for different regions.

Concerning the deposition of 7 µm aerosol in a transitory simulation, total and regional DE were analysed at various injection times (Fig. 6a). In the upper regions of the model (oral cavity, larynx and trachea) DE increased with increasing injection time, reaching the maximum value at the inspiratory peak. It rapidly decreased during the deceleration phase (1–2 s) of the inhalation cycle. Particles mostly deposited at the larynx walls, where the effect of the laryngeal jet combined with geometrical features determined a peak value of DE equal to 15.07% (${t}_{inj}=1$ s). At the 2nd and 3rd bifurcations, DE reached the maximum value at ${t}_{inj}=0.6$ s, while injecting particles at ${t}_{inj}=0.8$ s seemed to be more efficient concerning the 1st (main) bifurcation. Injection at ${t}_{inj}=0.8$ s was the most efficient strategy also considering the amount of particles deposited in the whole model: total DE at ${t}_{inj}$ this was equal to 34.78%, which is 20.33% larger than the one observed in steady simulations.

The unsteady nature of the airflow improved TB deposition, leading to a total DE of 18.44% + 4% than the one observed under steady-conditions; detailed deposition is reported in (Fig. 6c).

The percentage of particles leaving the terminal branches at various injection times is presented in Fig. 6b. The number of particles escaped from the lower (RLL, LLL) lobes is larger than that of the particles leaving the upper and medium lobes (RUL, RML, LUL). Maximum percentages were reached at ${t}_{inj}=0-0.2$ s, except for the RML lobe, where more particles left the RML when injected at ${t}_{inj}=0.8$ s. On the other side, particles injected during the deceleration phase (1.4–2 s) mostly floated through the domain so that deposition or escape data were not observed.

**Figure 7:**
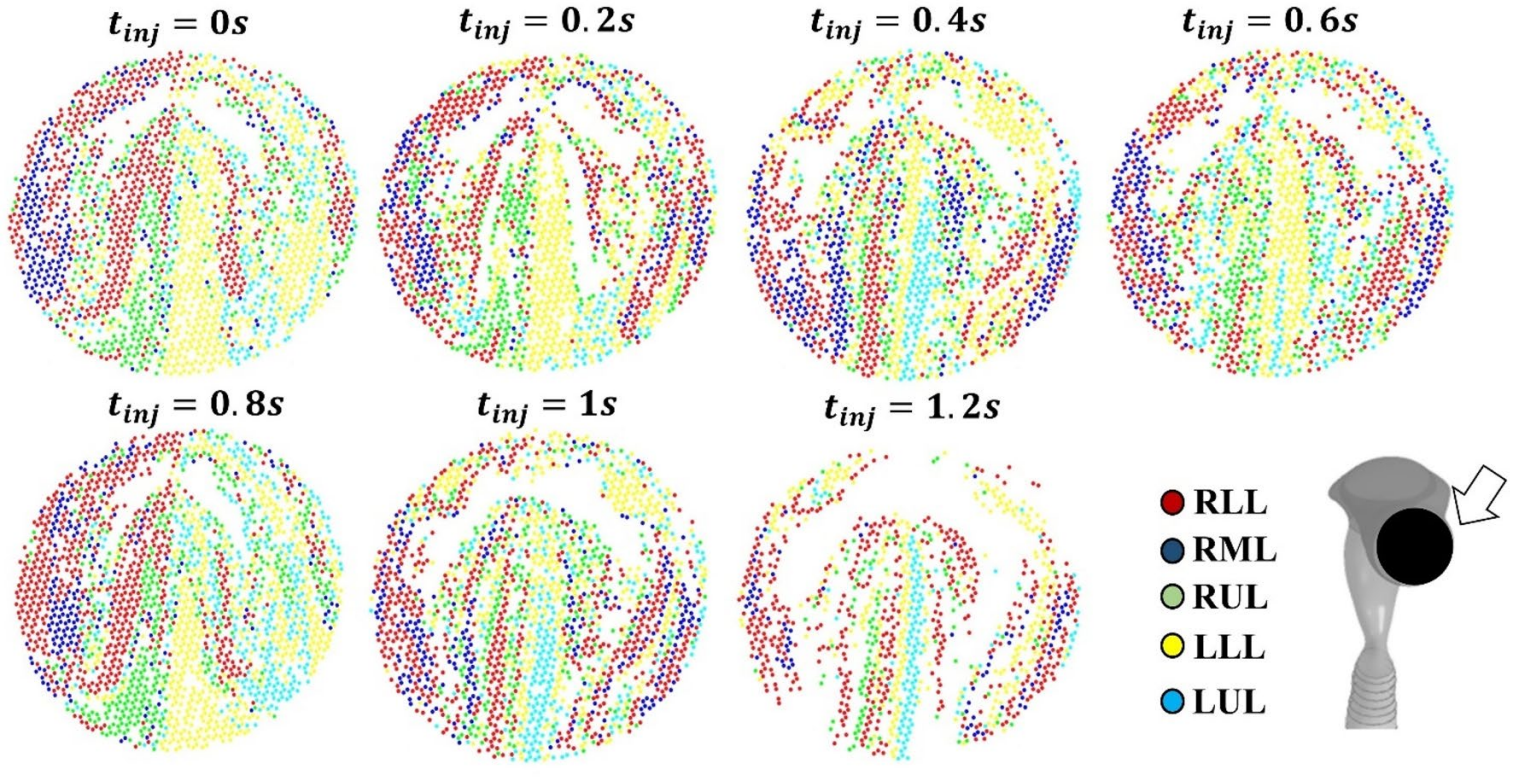
Transient simulation: 7 µm particle positions plot on the inlet surfaces of the mouth. Dots are aerosol particles, in the position where they are at their entrance in the model, at each indicated injection time. Colours indicate the lobes where aerosol exits from, empty regions are related to particles that either start from that position and deposit on the model internal surfaces, or remain suspended in the volume of the aerial way. (Matlab 2016-www.mathworks.com).

Considering the initial homogenous distribution of particles injected at the oral inlet, Fig. 7 shows particles exiting from different lobes with different colours. Particles leaving the same lobe were closed to each other, thus forming a layered distribution which evolves in time.

## Discussion

In the present work, a 3D characteristic model of the airway tree extending from the oral cavity to the 6th generation was developed to evaluate airflow dynamics and aerosol deposition under sedentary breathing physiologically conditions. Morphometric dimensions are consistent with physiological data available in the literature, extracted from in-vitro preparation of lung casts combined with CT technique.

The model was validated by comparing both airflow and pressure distribution through the lobes under steady state conditions of simulation, with physiological values reported in the literature^9,16,41,42^. Steady state simulations lay the foundations of further investigations, by the implementation of a robust transitory simulation model.

Highly-unstable phenomena were also correctly captured by the model. The laryngeal jet effect propagates downstream of the trachea (Figs. 1, 2, 3) with secondary two-vortex structures, proving that the reduced-CSA glottis is the dominant site of production of separation zones^14,15,18^, then the vortex rapidly dissipates downstream the trachea down to the 1st bifurcation.

These results confirm the morphometric accuracy of our model and the importance of including anatomic details like the curvature of the upper airways, the curvatures of bronchi and their branching angles.

The unsteady model also proved to be realistic in terms of particle deposition analyses. DE of particles inhaled under sedentary breathing conditions showed good agreement with results from previous studies^15,19,28–31^. Best fitting was provided for 7 µm particles, especially at the 1st and 2nd bifurcation (Fig. 5). Deposition in trachea shows to be less than how expected from other studies. This trend may be related to the presence of cartilaginous rings on the trachea walls, acting on the formation of boundary layer. Total deposition in transient conditions is higher than the results obtained in steady: such result points out the necessity to consider transient simulation to get a better description of the phenomenon.

Concerning the deposition of 7 µm particles, the sinusoidal airflow pattern showed a major effect on particles behaviour. The inhalation of the same particles sample at different time points led to different results in terms of both regional and total DE. The acceleration phase (0.6–1 s) proved to be the more efficient, especially on the laryngeal and 3rd bifurcations walls. Furthermore, considering the complete breathing cycle, deposition of particles inhaled in a transient regime was enhanced by 4% with respect to the constant airflow (steady) situation (Fig. 6c). Considering the injection time with the best performance, total DE was even enhanced by 20%. The improved deposition at bifurcation walls can be used to enhance the efficacy of therapeutic treatments in these regions.

Concerning particle trajectories, the distribution shown in Fig. 7 indicates that injections during the first breathing phases (0–0.2 s) drive aerosol to deep regions, so that particles leave the model. The number of particles escaped from the lower lobes (RLL, LLL) is larger than that of the particles leaving the upper and medium lobes, but there is no link between the initial position and the lobe that they escape from. Although it is impossible to predict the trajectories of particles based on their initial position, the model predicts a high percentage of 7 µm particles escaped: this aerosol is available for therapeutic deposition in deeper bronchioles of the human airways. Treatments of pathologies related to trachea are instead not optimized by this injection configuration but, based on these results, may benefit from an increase in the aerosol size.

About the modelling approach, the description of particles/droplets deposition, in conditions of laminar or quasi-transitional flow, is an issues common to a number of technical fields. Due to these heterogeneity of applications, it is not unusual to find different numerical settings, derived and dedicated to the geometrical and fluid dynamic conditions under investigation, but non-directly comparable one to each other. About this, in the field of T-junction fluid dynamics, the particles deposition on surfaces can be described by a different *St* formulation that represents the ratio of particle inertia to the fluid drag force^44^. Such a representation may fit better to predict particles deposition in bronchi, offering the same trend but higher values of St than the one considered in this paper. On the other hand such results would not be directly comparable to data available in the reference literature. Over the validation of a model and the comparison of new morphological and physiological conditions, further investigations on adjoining modelling solutions may consolidate the knowledge and the prediction of particles/droplets deposition in bronchi.

Our results shows the importance of including the transient behaviour of a physiologically breathing cycle to evaluate particle trajectories and deposition under sedentary conditions. Constant airflow simulations underestimate both local and total particle DE. Our model could be used to assess the efficacy of therapeutic aerosol treatment in different regions of human airways down to the 6th generation. Its applicability could be extended to different breathing conditions and to different particle types.

## Conclusions

The present 3D model of the respiratory tree contains characteristic geometric details of the oral cavity, pharynx, larynx and TB tree down to the 6th generation. Morphologic and morphometric features have been carefully reproduced in the design of bronchi around the bifurcation and of the cartilaginous rings in trachea. That guarantees a physiological airflow distribution through pulmonary lobes and realistic TB pressure drops for an average adult male patient. Transient simulation of a complete breathing cycle allowed us to describe highly-unstable phenomena like the formation of a laryngeal jet and recirculation patterns and investigate the resulting aerosol deposition. Aerosol DE was analysed at various injection times and compared with value observed under constant airflow rate. The number of particles leaving each lobe was also assessed. Our results show the importance of including a transient physiologically breathing cycle on aerosol deposition analyses. Furthermore the results indicate that there not relation between the position of the inhalation across the mouth and the deposition lobe, because the vorticity in the glottides and trachea sections mix the trajectories on the transversal plane.

In conclusion, our 3D model can be used to correctly analyse airflow and aerosol behaviour for an average adult male. The proposed model was developed to evaluate airflow dynamics under sedentary breathing conditions. It is suitable for scalability to allow analysis of different breathing pattern (i.e. light breathing, heavy breathing), different subjects (i.e. average paediatric or elderly, male or female) or pathological airways conditions. Knowledge of DE in specific regions may guide the choice of the droplet and injection settings, in order to target well confined pathologies. Moreover the presented model can become a tool to test the behaviour of particles differing in size and rheology. Future research directions could include extension of the model to deeper generations in order to evaluate DE further down to the bronchiole regions. Deeper anatomical structures, could be an improvement only if the reliability of results is preserved: the deformability of further bifurcation structures may affect the definitions of boundaries along the breath cycle so it should be considered in the definition of a more extended model.

Computational simulations tools have proved to be able to show flows and particles depositions, assemble data from several geometrical features and time instants into a usable frames, as tools for the design of protocols and devices for aerosol administration.

## Materials and methods

### Anatomical model

**Figure 8:**
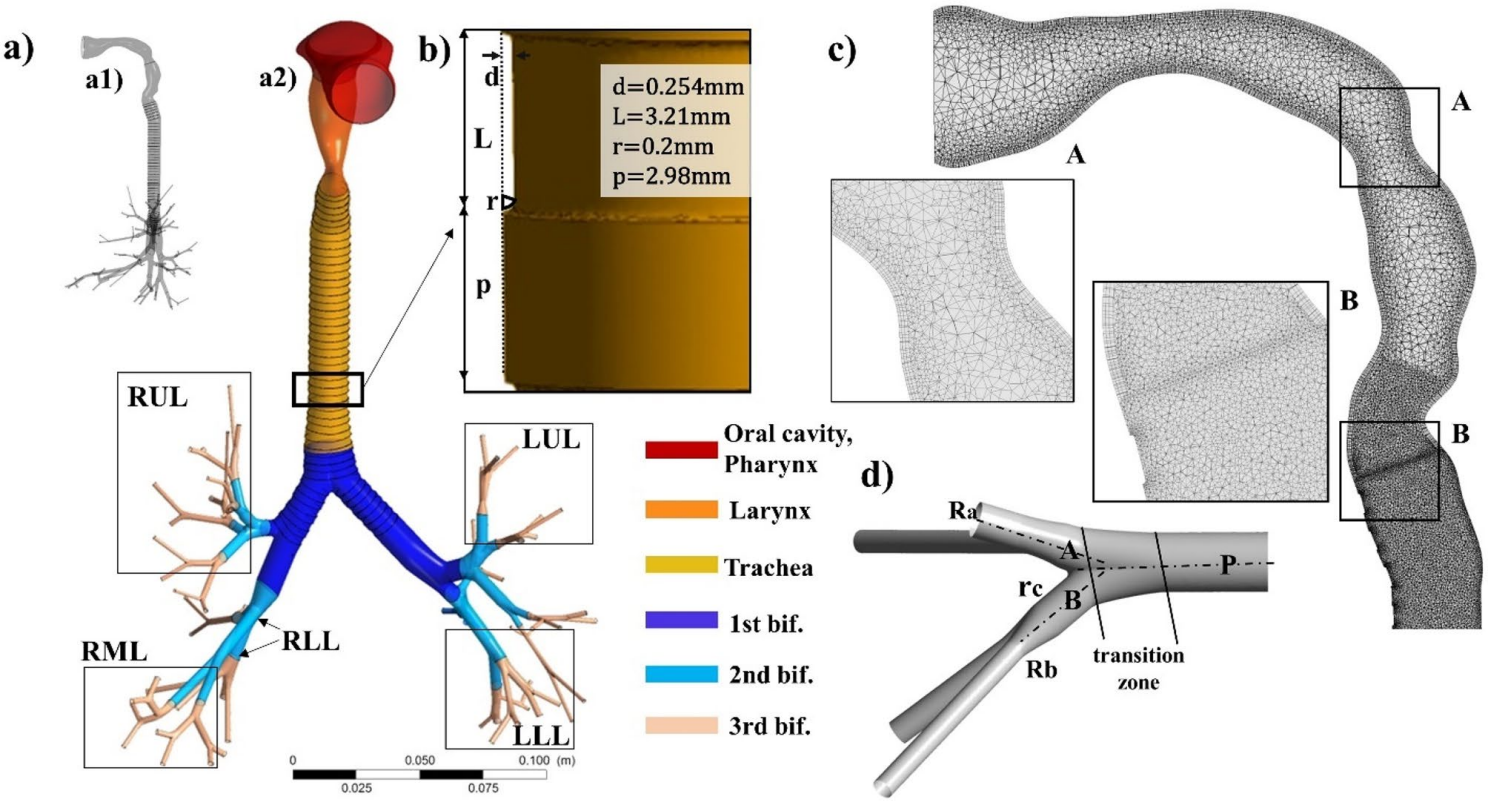
3D realistic airway tree model in details: (**a**) lateral and frontal view of the 3D realistic airway tree created and the correspondent lobes: right-upper (RUL), right-medium (RML), right-lower (RLL), left-upper (LUL) and left-lower (LLL). (**b**) Dimensions of the cartilaginous rings, based on in-vitro observations of Russo et al.^39^; *p* and *L* are the step and the width, respectively, *d* is the depth of the ring and r is the fillet radius. (**c**) Representative computational grid of the model.

The 3D airway model contains a characteristic geometry of the oral cavity, throat and TB tree down to the 6th generation, including physiological bifurcations and cartilaginous rings (Fig. 8a).

The reconstruction of the TB tree was based on coordinates and dimensions of the Schmidt digital model^8^, (see “Supplementary Materials” online), and obtained with the computer-aided design (CAD) software Solidworks 2016 (Dassault Systèmes Solidworks Corp., Waltham, MA USA). A special attention was given to the design of branches sizes and bifurcations. The relationship between parent (P) and daughter branches (A, B) follows Eq. (2):

2 $${d}_{P}^{{x}_{n}}={d}_{A}^{{x}_{n}}+{d}_{B}^{{x}_{n}}$$

where ${\mathrm{d}}_{\mathrm{P}}^{{\mathrm{x}}_{\mathrm{n}}}$ is the parent branch diameter, ${d}_{A}^{{x}_{n}}$ and ${d}_{B}^{{x}_{n}}$ are the daughter ones and ${x}_{n}$ is the exponent which determines the flow conditions in the *n*th bifurcation.

Section A shows the prismatic layers created near the larynx wall, Section B shows the finest tetrahedral element of the tracheal mesh. (d) Model of physiologically realistic bifurcation based on Heistracher et al.^22^; ${r}_{c}$ is the carinal radius of curvature, ${R}_{A}$ and ${R}_{B}$ are the daughter radii and ${c}_{c}$ is the ratio of carinal curvature ratio. (CFD-Post v16, ICEM CFD v15-Ansys, www.ansys.com).

To reproduce physiologically characteristic bifurcations (Fig. 8d), every parent segment was split into two daughter tubes with a transitional zone starting from the 80% of its axial length. Then, each transition zone was smoothed defining the characteristic carinal radius of curvature ${r}_{c}$ through the equation:

3 $${r}_{c}=\left({R}_{A}+{R}_{B}\right){ c}_{c}$$

where ${R}_{A}$ and ${R}_{B}$ are the daughter radii and ${c}_{c}$ is the ratio of carinal curvature ratio equal to 0.1^23^. The tracheal section was modelled as a circular tube with a diameter equal to 1.56 cm, cut by a posterior plane at 0.65 cm from the centre^45^ thus reproducing the tracheal typical C-shape due to the presence of the pars membranacea^46^. As shown in Fig. 8b, tracheal and main bronchi walls were enriched with cartilaginous rings, whose dimensions are consistent with in-vitro observations of Russo et al.^39^. The rings arrangement was made regularly perpendicular to the axial branch direction, while the rings orientation was adapted in the transitional zones to maintain their parallelism with the curvature radii.

A characteristic model of extra thoracic airways was created by shaping the oral cavity, pharynx and larynx as elliptical tubes (see “Supplementary Materials”, Fig. S1). Geometrical parameters of each section were extracted from Cheng’s silicone model^29^. Then hydraulic diameters and cross sectional areas (CSA) were scaled in order to fit Schmidt realistic model of TB tree.

### Grid generation

The 3-D characteristic airway model was divided into 24 bodies according to size and anatomical region: oral cavity and pharynx, larynx, trachea, main bronchi bifurcation (1st bif.), second (2nd bif.) and third (3rd bif.) generations. There are 64 peripheral small airways. ICEM CFD v15 software (Ansys Inc., Canonsburg, PA, USA) was employed to discretize the model with tetrahedral volume elements, triangular superficial elements and a multi-layer prism mesh to solve the high velocity gradient expected at the upper airways and larynx walls (Fig. 8c). The best size of the grid to solve this problem (trade-off between accuracy of results and calculation time) was selected by a sensitivity analysis (see “Supplementary Materials”, Fig. S2) and results in a mesh of 9,826,620 tetrahedral elements and three boundary layers of hexahedral ones.

### Numerical solutions and settings—airflow

Numerical simulations were performed with the commercial CFD code Fluent v16 (ANSYS, Inc., Canonsburg, PA, USA) on an Dell T630 Workstation with 64 Gb RAM and two 1.8 GHz CPUs. Air was assumed to be an incompressible and Newtonian fluid with constant density $\rho =1.225$ kg/m^3^ and viscosity $\upmu = 1.7894 \times 10^{ - 5} \;{\text{kg/m}}\;{\text{s}}$. The continuity and Navier–Stokes equations (Eqs. 3 and 4) for the airflow phase were discretized with second order accuracy in time and space under both steady-state and transient conditions, in laminar regime. The Green-Gauss Node-Based algorithm was utilized for pressure–velocity coupling:

4 $$\frac{\partial \rho }{\partial t}+\nabla \left(\rho \overrightarrow{u}\right)=0$$

5 $$\uprho \left\{ { \frac{{\partial \vec{u}}}{\partial t} + \left( {\vec{u}\nabla } \right)\vec{u} } \right\} = - \nabla P + \mu \nabla^{2} \vec{u} + \rho \vec{f}$$

Transient CFD simulations were performed to reproduce a complete sedentary breathing cycle. Such a cycle was modelled with a sinusoidal curve 4 s long, with inhalation and exhalation phases of the same duration^14^. Airflow had mean and peak values equal to 6 L/min and 9.42 L/min, respectively.

A user-defined function (UDF) was implemented to impose a time-dependent, sinusoidal velocity profile at the *i*-the outlet of the model, through the formula:

6 $$u_{i} \left( t \right) = u_{i,max} \;{\text{sin}}\left( {\frac{2\pi t}{T}} \right)$$

where ${u}_{i,max}$ is the maximum velocity at the *i*th outflow extracted from steady-state solutions (see “Supplementary Materials”), *T* is the breathing period and *t* is time. No-slip boundary conditions and zero pressure were assumed at airway walls and at the oral inlet, respectively.

### Numerical solutions and settings-particles

Particle transport equations were solved using DPM available in Fluent v16 (ANSYS, Inc., Canonsburg, PA, USA), in combination with user-defined injection patterns and boundary conditions. DPM (see “Supplementary Materials”) tracks individual particles as they move through the flow using a Lagrangian approach. The Navier–Stokes equations for the continuous and discrete phases were solved separately in the steady-state condition, while they were solved together in the transient regime.

Particle deposition is usually assessed as a function of particle Stokes number St:

7 $$St=\frac{{\rho }_{p}{d}_{p}^{2}U{C}_{c}}{36\mu {R}_{0}}$$

where ${\mathrm{R}}_{0}$ and $\mathrm{U}$ are the average radius and the mean velocity of the airflow in the parent branch, respectively and *C*_*c*_ is the Cunningham slip correction factor defined by Hinds et al.^32^. St is typically considered to express the ratio between the particle stopping distance and a characteristic dimension of an obstacle. Thus at large Stokes numbers, particles may deviate from fluid streamlines and impact on obstacle surface, while at small numbers they tend to follow fluid streamlines^15,32^. The particles simulated in our analyses had constant diameters $({ 3\le \mathrm{d}}_{\mathrm{p}}\le 25$ µm), which is the typical range of aerosol treatment. Density and viscosity were set equal to water-liquid ones, thus $\rho =998.2$ kg/m^3^ and viscosity $\mu =0.001$ kg/m s and inert behaviour was considered. ${C}_{c}$ was set equal to 1, in accordance with observations reported in Hinds et al.^32^, and Tu et al.^47^. The injection pattern consisted of 2720 particles homogeneously distributed on the oral surface, forming a disk with a diameter equal to 2.16 cm at a constant distance from the oral boundary. The boundary conditions for the equations governing particle motion included zero initial velocity, deposition when the particle centre reaches the wall and escape conditions at the 64 outlets. Four different diameters equal to 3, 7, 10 and 25 µm were considered to assess the effect of particle size on DE under a constant airflow rate (6 L/min). Numerical predictions of total and regional DE were compared with both empirical formulations and experimental evidences available in the literature^3,15,29–31^. Tracheobronchial deposition data were validated with the empirical results of Zhou et al.^28^, based on in vitro experiments of polystyrene latex particles. The general formulation used for DE calculation was:

8 $$\mathrm{DE}=1-\mathrm{exp}(-\mathrm{a}*{\mathrm{St}}^{\mathrm{b}})$$

where a and b are empirical constants, which differ according to the airway of interest. Upper airways DE were compared with the experimental evidences found in Cheng et al.^29^ on oral airway silicone replicas, defining DE as a function of the impaction parameter $\mathrm{IP}=\uprho {\mathrm{d}}_{\mathrm{p}}^{2}\mathrm{Q}$:

9 $$\mathrm{DE}=1-\mathrm{exp}(-\mathrm{a}*\mathrm{IP})$$

DE of 7 µm particles was also analysed under unsteady condition. The same distribution of 2720 particles were injected at the oral inlet every 0.2 s during both ascendant [at 0, 0.2, 0.4, 0.6 and 0.8 s] and descendant parts [at 1, 1.2, 1.4, 1.6 and 1.8 s] of the inhalation phase. Furthermore, an in-house MATLAB code (MATLAB v9.2, The MathWorks, Inc., Natick, MA, USA) was implemented to calculate the amount of particles exiting from the 64 outlets of the model.

## Supplementary Information

Supplementary Information.

## Data Availability

The morphological model is based on LungSim—Multiscale simulation of human lung—https://simtk.org/projects/lungsim, a system engineered representation of the human lung based on multiscale imaging (Multidetector CT, micro-CT, microscopy). The use of data is coherent with the declared terms of use.
