# Supplementary material for: Computational fluid dynamic models as tools to predict aerosol distribution in tracheobronchial airways
Source: Sci Rep. 2021 Jan 13;11:1109. doi: 10.1038/s41598-020-80241-0 (PMC7806585; doi:10.1038/s41598-020-80241-0)
Supplement: Supplementary file 1 — Supplementary Information. [file 41598_2020_80241_MOESM1_ESM.pdf]

Computational fluid dynamic models as tools to predict aerosol distribution in tracheobronchial airways.

Claudia Atzeni, Gianluca Lesma, Gabriele Dubini, Maurizio Masi, Filippo Rossi, Elena Bianchi*

SM1
LungSim - Multiscale simulation of human lung - https://simtk.org/projects/lungsim
"The simulation software in this repository is a system engineered representation of the human lung based on multiscale imaging (Multidetector CT, micro-CT, microscopy). It allows to investigate dimensions and heterogeneities in geometric branching patterns, gas supply and diffusion capacities. The research being conducted with this tool provides insight into the evolutionary shaped design of the respiratory system, physical optimality and robustness against perturbations caused by the aging process or disease.
LungSim v1.2 is a self-installable file for MS Windows including a demonstration dataset. The models are part of the academic output of the researchers cited and should be referred to as such. The project received funding by DFG and NIH-NIBIB. Developers included in the various phases of LungSim's history are Andreas Schmidt, Thorsten Denhard, Stephan Zidowitz, Saloni Shah, Viraj Shah and Uday K. Thummalapalli.

SM2

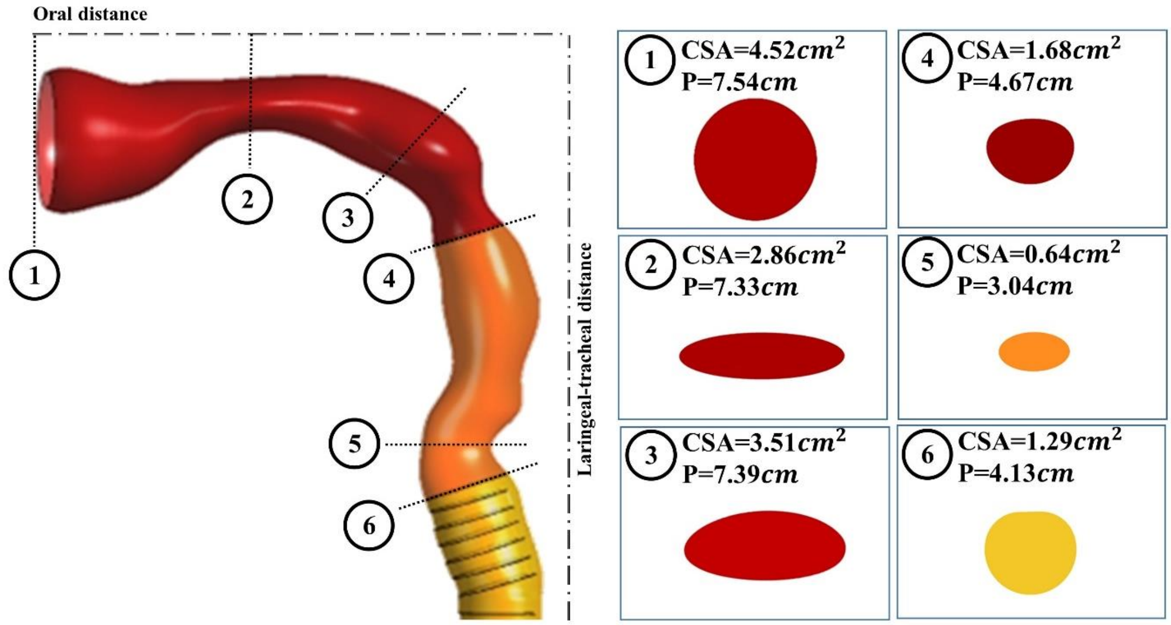

Figure. S1 Regions and cross sections of the respiratory system model Critical cross-sectional areas and perimeters of the upper airways model; r is the anatomical curvature radius of the throat, α is the orientation of the trachea with respect to the vertical axis (13). In addition, the trachea was oriented at an angle α=17° from the vertical axis to match CT-scan analyses by Xi and Longest and the anatomical curvature of the throat was reproduced by imposing a curvature radius r=3.27 cm 13. (CFD-Post v16 – Ansys, www.Ansys.com)

SM3
Our approach was based on the Octree-based method and Laplace smoothing to generate a high resolution surface mesh, while the volumetric elements were subsequently created with the Delaunay algorithm. Different grids consisting of 8,714,823 (Mesh 1), 9,826,620 (Mesh 2) and 10,496,004 (Mesh 3) cells were created to evaluate the grid size sensitivity for the flow solution.

A boundary layer size sensitivity was also performed to fully capture the highly complex velocity profile near the larynx-glottis walls, discretising the region at a constant distance of $\delta = 0.73\ mm$ from the upper airways and larynx walls with three and ten layers of prismatic elements, respectively.
Grid independency results in terms of velocity profile at the two sections A and B in the trachea. BL is the number of elements in the boundary layer.

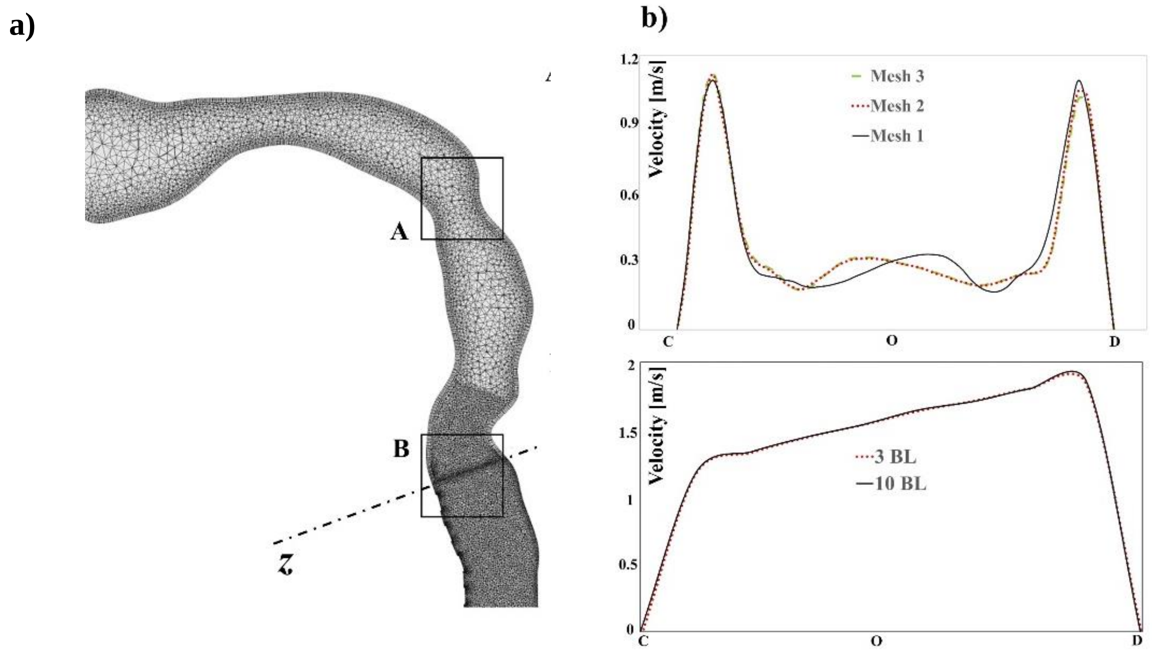

Figure S2 shows the velocity profile over the non-dimensional length in both tracheal and laryngeal sections. indicate that Mesh 1 grid was hardly sufficient to capture airflow fluid dynamics, while the increase of tetrahedral element size up to 10,496,004 cells (Mesh 3) did not change the results in terms of velocity profile in the most critical regions with respect to Mesh 2. Furthermore, three boundary layers proved to be sufficient to fully reconstruct the recirculation zones occurring at laryngeal walls. Hence, Mesh 2 with 9,826,620 tetrahedral elements and three boundary layers of hexahedral ones was adopted to perform the numerical simulations. (ICEM CFD v15 – Ansys , www.Ansys.com)

SM4 – preliminary simulations steady
Steady-state simulations were performed to evaluate airflow distribution through the model. A uniform velocity field was applied at the oral inlet with an average value of 0.2211 m/s. This value corresponds to a minute volume of 6 L/min, which is representative of sedentary breathing conditions for an average adult male (Guyton, 1977). No-slip boundary conditions were assumed at the airway walls, while constant zero-pressure was prescribed at the 64 outlets of the model. Steady-state solution of the flow field was assumed convergent when the residuals of the governing equations reached values <10^(-6).
Flow distribution through the airway model at steady state complies with physiological parameters.
About transient simulations : time step size was set to 5 ms and the maximum Fluent Courant number was 20. Transient solution was assumed convergent at each time step when the residuals of the governing equations reached values <10^(-4), in accordance with other studies found in literature (Elcner et al., 2016).

SM5
The motion of spherical liquid particles suspended in air is governed by Newton's Second Law:

$$\frac{d\overrightarrow{u_{p,i}}}{dt} = \sum \vec{F}_p \qquad (6)$$

where $u_{p,i}$ is the velocity of the i particle and $\vec{F}_p$ the force acting on it.

Considering small particles, Reynolds numbers $Re_p = \frac{\rho d_p |u_{p,i} - u_i|}{\mu} \ll 1$, a large density ratio $\frac{\rho_p}{\rho} \gg 1$ and particle diameter $d_p > 1\mu m$, most of the known particle forces other than drag force can be neglected (Zhang, 2002), thus Eq. (6) can be simplified as follows:

$$\frac{d\overrightarrow{u_{p,i}}}{dt} = F_D(\overrightarrow{u_i} - \overrightarrow{u_{p,i}}) \qquad (7)$$

where $u_{p,i}$ and u_i are the components of particle and fluid velocity in the $i - th$ position, respectively. For spherical particles, the drag force per unit mass is defined as:

$$F_D = \frac{18\mu}{\rho_p d_p^2 C_c} \frac{C_D Re_p}{24} \quad (8)$$

where C_D is the drag coefficient defined by Morsi and Alexander (Morsi, 1972), d_p is the particle diameter and C_c is the Cunningham slip correction factor defined by Hinds et al. (Hinds, 1999).

With particle Reynolds numbers $Re_p < 1$ and very small Stokes numbers ($St < 0.25$), several authors found that particle motion can be basically achieved with a first-order correction to fluid element motion equation (Comer 2000) (Zhang 2001; Zhang 2002). For example, Comer observed that the largest influence of the Cunningham slip correction factor C_c was a 2% change in particle DE at St = 0.24 (Comer, PhD thesis publication). In this sense, C_c is used for particles in the nanometric range, where the primary phase cannot be considered continuous (Tu, 2013). Furthermore, the correction to Stokes law for non-rigid spheres as water droplets is generally insignificant (Hinds 1999; Tu 2013; Fluent Guide, Fluent 16, Ansys Inc.).

SM6

**region**	**particle diameter [μm]**			
	**3**	**7**	**10**	**25**
Oral cavity. Pharynx	0.33%	0.29%	0.40%	3.86%
Larynx	**5.29%**	**6.29%**	**8.75%**	**53.24%**
Trachea	0.77%	1.07%	2.43%	14.34%
1^{st}bifurcation	1.47%	1.43%	1.51%	0.59%
RUL	0.44%	0.40%	1.51%	7.68%
RLL	1.88%	2.13%	3.71%	5.74%
LUL	0.29%	1.03%	1.14%	1.76%
LLL	0.99%	1.8%	3.05%	4.04%
**Total DE**	**11.47%**	**14.45%**	**22.51%**	**91.25%**

Table S1. Deposition efficiency (DE) of 3-25μm particles injected under steady conditions (Q=6 L/min). RUL and RLL are the right upper and lower lobes, LUL and LLL are the left upper and lower ones. Grey cells represent the maximum DE of each region of the model, bold font highlights the maximum DE for various particle diameters.
